# Pediatric Post-COVID-19 Neuromyelitis Optica Spectrum Disorder: A Case Report

**DOI:** 10.7759/cureus.113264

**Published:** 2026-07-23

**Authors:** Julieth Bibiana Espinel-Porras, Laura Daniela Arenas, Victor Manuel Mora-Bautista

**Affiliations:** 1 Pediatrics, Universidad de Santander, Bucaramanga, COL; 2 Pediatrics, Clínica San Luis, Bucaramanga, COL; 3 Pediatrics, Universidad Industrial de Santander, Bucaramanga, COL

**Keywords:** aquaporin 4, azathioprine, covid-19, neuromyelitis optica, pediatrics, plasmapheresis, rituximab, transverse myelitis

## Abstract

Neuromyelitis optica spectrum disorder (NMOSD) is a rare autoimmune astrocytopathy affecting the central nervous system, particularly the optic nerves and spinal cord. Post-infectious triggers, including SARS-CoV-2 (COVID-19), have been increasingly recognized as potential catalysts for this immune-mediated condition.

We report the case of a nine-year-old boy who developed AQP4-IgG-positive NMOSD following confirmed SARS-CoV-2 infection. The patient's clinical onset began 1.5 months prior to admission with a seven-day episode of intractable vomiting, indicative of area postrema syndrome (APS). He subsequently presented with a 20-day history of progressive gait weakness, dysarthria, and complex ocular and cranial nerve deficits. Neurological examination revealed a right one-and-a-half syndrome, bilateral sixth cranial nerve paresis, left peripheral facial paresis, profound bulbar dysfunction (including a weak gag reflex and bilateral 12th nerve paresis), lower limb weakness (4/5), gait ataxia, and bilateral extensor plantar responses. Magnetic resonance imaging (MRI) demonstrated extensive central nervous system involvement. Brain MRI revealed typical hyperintense lesions in the periventricular white matter, thalamic-hypothalamic area, midbrain, medulla, and area postrema, alongside bilateral retrobulbar optic nerve enhancement. Spinal imaging confirmed longitudinally extensive transverse myelitis (LETM) spanning the C1-C6 and T2-T6 levels, featuring central gray matter involvement (H-sign) extending into the lateral columns. Cerebrospinal fluid was largely unremarkable. Serological testing confirmed the diagnosis with positive aquaporin-4 (AQP4)-IgG antibodies (cell-based assay (CBA)), positive antinuclear antibodies (ANAs) (1:80), and positive COVID-19 IgG antibodies, while anti-MOG antibodies were negative. Acute targeted immunotherapy was initiated with high-dose intravenous methylprednisolone (30 mg/kg/day for five days). While this achieved complete resolution of his gait abnormalities, visual impairment persisted. He subsequently underwent five sessions of therapeutic plasmapheresis as rescue therapy, resulting in full visual and neurological recovery. The patient was discharged on a maintenance regimen of oral azathioprine (2 mg/kg/day) and biannual intravenous rituximab. A four-month follow-up MRI confirmed dramatic radiologic improvement, with complete resolution of the spinal cord abnormalities.

This pediatric case highlights post-COVID-19 NMOSD as a severe but highly treatable neurological emergency. It underscores the critical need for rapid diagnosis through detailed clinical recognition (including preceding APS), advanced radiographic imaging, and targeted serologic evaluation, as well as the efficacy of plasmapheresis for steroid-refractory symptoms to prevent irreversible disability.

## Introduction

Neuromyelitis optica spectrum disorder (NMOSD) is a rare autoimmune demyelinating disorder characterized by extensive transverse myelitis, optic neuritis, and area postrema syndrome (APS), a clinical entity defined by intractable nausea, vomiting, or hiccups (singultus) lasting for at least 48 hours. Its prevalence ranges from 0.3 to 4.4 cases per 100,000 adults, with an incidence of 0.05-0.4 per 100,000 per year and a marked female predominance (9:1 ratio), typically presenting in the third decade of life [1-3]. This condition belongs to the spectrum of antibody-mediated central nervous system disorders, alongside myelin oligodendrocyte glycoprotein antibody disease (MOGAD) and multiple sclerosis (MS). Although clinical overlap led to the historical consideration of NMOSD and MOGAD as MS variants, distinct clinical, serologic, imaging, and prognostic features established them as independent entities by 2023, with MOGAD being more frequent and carrying a better prognosis [2-5].

In Colombia, NMOSD onset occurs between the ages of 40 and 60 years, with a prevalence of 5.3 per 100,000 inhabitants - among the highest in Latin America - and a 3:1 female-to-male ratio [6,7]. While susceptibility genes remain unidentified, human leukocyte antigen (HLA)-DPB1*0501 associations have been reported in Asian populations. Both infections and vaccines (yellow fever, influenza, hepatitis B, HPV, and COVID-19) have been implicated as triggers [8].

NMOSD pathogenesis involves immunoglobulin G antibodies against aquaporin-4 (AQP4) water channels on astrocytes in the spinal cord, optic nerves, pia mater, and ependymal surfaces. Complement activation triggers neutrophil and eosinophil degranulation, immune complex deposition, axonal injury, and neuronal death, facilitated by blood-brain barrier-deficient regions such as the area postrema [1,5]. Conversely, MOGAD features predominantly extrathecal autoantibody production (though intrathecal synthesis may occur). Interleukin-6 drives MOG-IgG-secreting plasmablasts, which cross the blood-brain barrier, target oligodendrocytes, and trigger complement-mediated damage alongside CD4+ T-cell activation (whereas CD8+ T cells predominate in MS) [5].

The 2015 International Consensus Diagnostic Criteria for NMOSD require optic nerve involvement (unilateral or bilateral acuity/motility impairment) plus spinal cord involvement, intractable nausea/vomiting, headache, or sensory loss. Cerebrospinal fluid typically lacks oligoclonal bands (<20% of cases), though hyperproteinorrhachia (>40 mg/dL) or pleocytosis (>50 cells/field) may occur. Magnetic resonance imaging (MRI) demonstrates spinal cord edema with contrast enhancement in the optic nerves, brainstem, diencephalon, hypothalamus, and periventricular regions [2]. Since 2023, live-cell assays for AQP4 and MOG antibodies have been mandatory [3,5].

Pediatric cases (5% to 10%) mirror adult presentations, radiology, and laboratory findings, permitting use of identical diagnostic criteria [1,9]. Since 2020, increasing reports have documented an association between NMOSD and SARS-CoV-2 infection [10,11] and COVID-19 vaccination [11,12], with AQP4+ mechanistic links established by 2023 [13]. Vaccine- and infection-related cases share similar clinical courses [11], though pediatric cases remain scarce [10,14]. Post-COVID-19 or post-vaccination MOGAD exceeds NMOSD (2:1 ratio), although both constitute <1:1,000,000 events, predominantly affecting adults with encephalitis. MOGAD maintains a superior prognosis, whereas pediatric NMOSD appears more treatment-refractory [14,15].

We present a pediatric post-COVID-19 NMOSD case, underscoring its inclusion within SARS-CoV-2 immunologic complications - a highly morbid yet fully treatable entity when recognized early.

## Case presentation

Clinical summary

We present the case of a nine-year-old male who developed subacute progressive bulbar and motor dysfunction, characterized by right one-and-a-half syndrome, facial paresis, and gait weakness, preceded by a seven-day episode of intractable vomiting (APS). Subsequent neuroimaging and serological testing confirmed AQP4-IgG-positive NMOSD with longitudinally extensive transverse myelitis (LETM) in a post-COVID-19 context. The patient achieved full neurological recovery following targeted acute immunotherapy and plasmapheresis.

Detailed case description

A nine-year-old male schoolboy from Piedecuesta, Santander (Colombia), presented with a 20-day history of progressive gait weakness, dysarthria, and facial edema, associated with fever on the previous day. He had received two doses of the CoronaVac® vaccine two years prior. No other relevant medical history was documented. Ophthalmologic examination revealed normal fundoscopy. Due to the acute clinical setting and the retrospective nature of the report, formal visual acuity metrics, color vision (Ishihara), visual fields, and pupillary reflexes were not documented. Extraocular motility evaluation revealed a right one-and-a-half syndrome (right conjugate horizontal gaze palsy plus right ipsilateral internuclear ophthalmoplegia, meaning the eye on the affected side cannot look horizontally in either direction, and the other eye cannot adduct, though it can abduct with nystagmus), with bilateral sixth cranial nerve paresis, right medial gaze palsy, and mild left medial gaze paresis, without vertical gaze impairment. Additional findings included left peripheral facial paresis, a weak gag reflex, poor palate elevation, bilateral twelfth cranial nerve paresis, lower limb weakness (gluteus maximus, hamstrings, quadriceps) with muscle strength of 4/5, mild hyperreflexia in the lower limbs (+++/-++++), bilateral extensor plantar response, gait ataxia, normal dysdiadochokinesia, and mild bilateral action tremor. Upper limb strength was preserved.

Brain computed tomography was normal. Initial laboratory findings are summarized in Table 1, revealing normal cerebrospinal fluid with isolated mild neutrophilia, lymphopenia, and alanine aminotransferase (ALT) elevation.

**Table 1:** Initial laboratory findings with pediatric reference ranges Notes: Oligoclonal bands: negative. Dengue: NS1+, IgM-, IgG-. COVID-19: IgG+, IgM-. Other serologies: negative (adenovirus, EBV, CMV, syphilis, HIV). AST: Aspartate aminotransferase; ALT: Alanine aminotransferase; CMV: Cytomegalovirus; EBV: Epstein-Barr virus; HIV: Human immunodeficiency virus; CBC: Complete blood count; CSF: Cerebrospinal fluid

Parameter	Patient Value	Reference Range (Pediatric)
CSF		
Leukocytes	0 cells/μL	0-5 cells/μL
Glucose	62.2 mg/dL	40-70 mg/dL
Protein	23.4 mg/dL	15-45 mg/dL
CBC		
Leukocytes	5,130/μL	4,500-13,500/μL
Neutrophils	75.6%	35-65% ↑
Lymphocytes	14.5%	25-55% ↓
Hemoglobin	14.7 g/dL	11.5-15.5 g/dL
Hematocrit	43.1%	35-45%
Platelets	181,000/μL	140-450 x 10³/μL
Chemistry		
AST	51.7 U/L	15-60 U/L
ALT	47.1 U/L	5-45 U/L ↑
Sodium	136 mmol/L	135-145 mmol/L
Potassium	4.9 mmol/L	3.5-5.5 mmol/L

**Figure 1:**
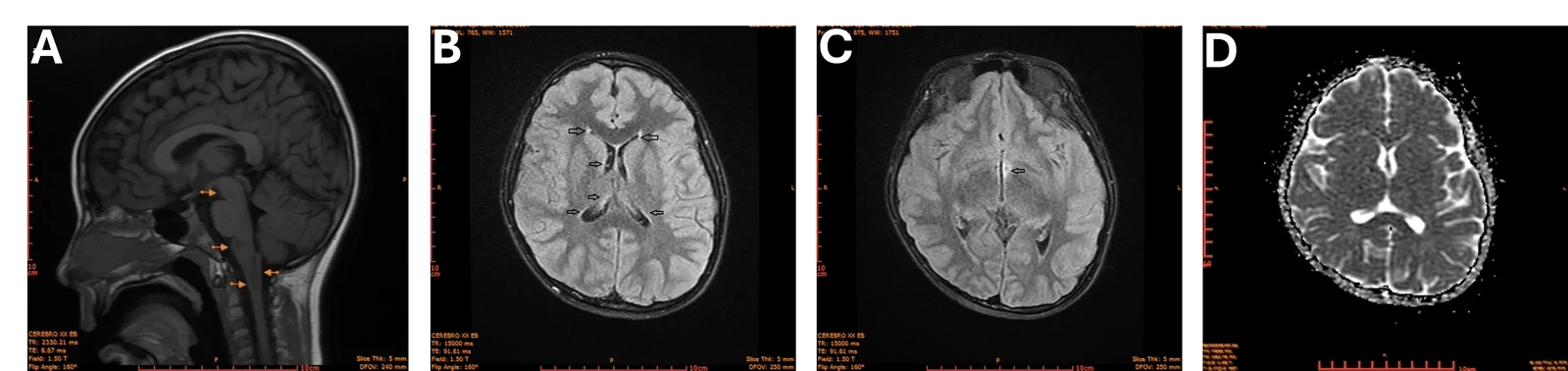
T1 and T2 FLAIR MRI sequences demonstrating characteristic NMOSD neuroanatomical distribution (A) T1-weighted sequence highlighting hypointensities (arrows) in the periaqueductal region (midbrain), anterior and posterior medulla, and dorsal medullary region (area postrema) extending to the cervicomedullary junction. T2-FLAIR sequences demonstrate representative hyperintense lesions (arrows) in the periventricular white matter (B) and the diencephalic region, specifically the left thalamic-hypothalamic area (C). (D) Diffusion-weighted imaging (DWI) shows no restricted diffusion. This distribution of lesions is typical of NMOSD. NMOSD: Neuromyelitis optica spectrum disorder; MRI: Magnetic resonance imaging; FLAIR: Fluid-attenuated inversion recovery

**Figure 2:**
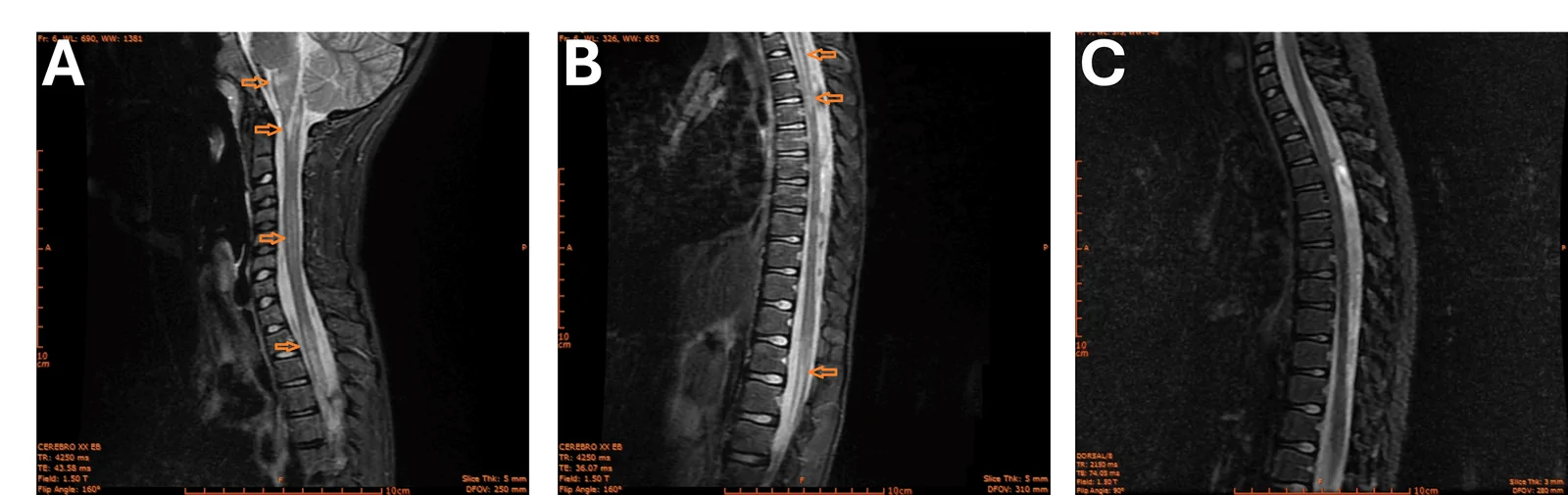
STIR MRI sequence (A) and (B) Baseline images. Hyperintensities are observed (arrows) in the medulla, periaqueductal area, and area postrema, along with involvement of the cervical and thoracic spinal cord (centromedullary). In panel B, an additional arrow identifies a hyperintensity extending into the conus medullaris. (C) Four-month follow-up. No spinal cord abnormalities are present. STIR: Short tau inversion recovery. A magnetic resonance imaging (MRI) technique that suppresses fat signals, enhancing the visualization of tissues and pathologies.

**Figure 3:**
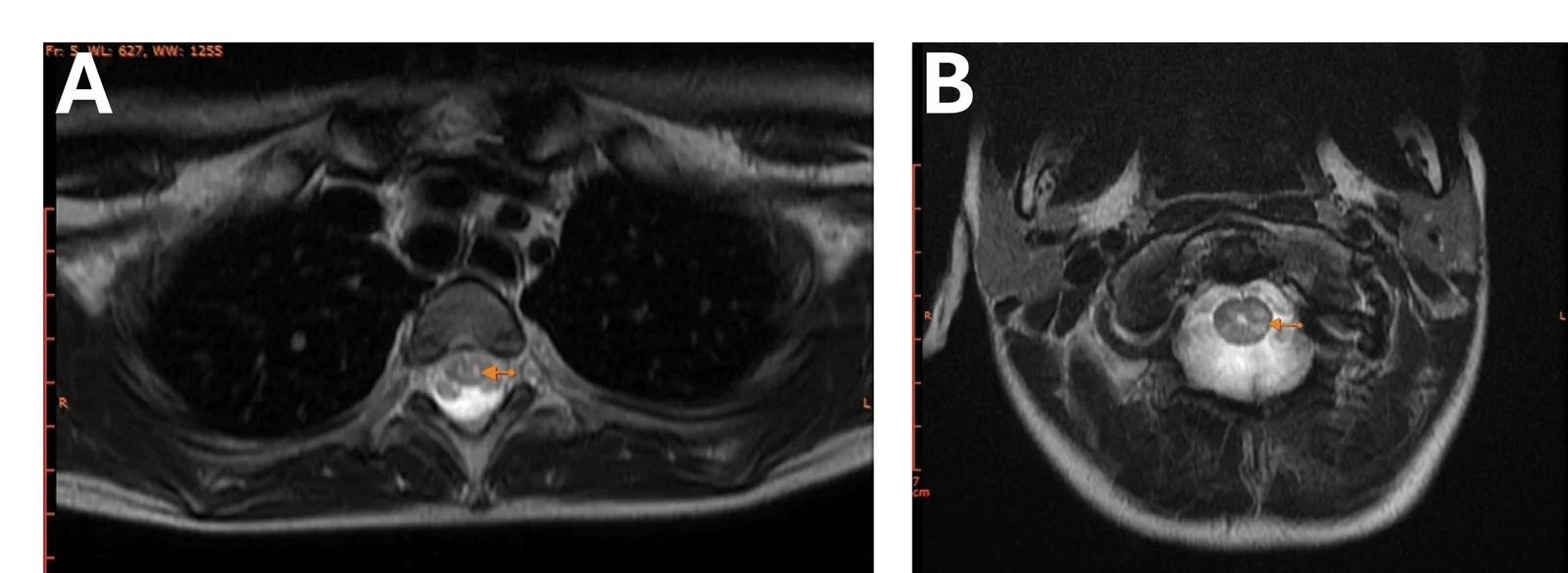
T2 sequence (A) D2 vertebral level. H-sign (arrow), representing a distinct T2-hyperintense signal pattern completely mapping the central gray matter, with bilateral extension into the adjacent white matter lateral columns, a finding more common in MOGAD than in NMOSD. (B) Cervicomedullary junction. Centromedullary and specific left lateral column signal involvement (arrow), which, along with the longitudinally extensive spinal cord involvement (Figure 2), is a pattern more typical of NMOSD. MOGAD: Myelin oligodendrocyte glycoprotein antibody disease; NMOSD: Neuromyelitis optica spectrum disorder

**Figure 4:**
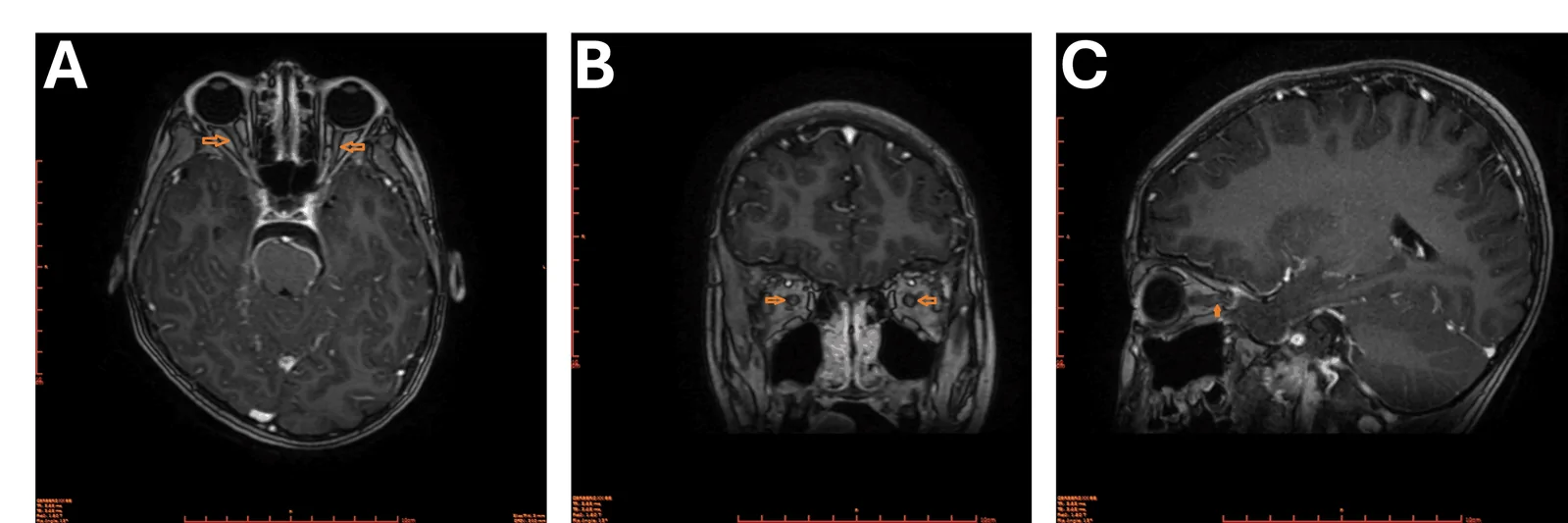
Contrast-enhanced brain MRI (A) Axial view. (B) Sagittal view. (C) Coronal view. Mild enhancement of the mid-portion of the optic nerves is observed across all three planes (arrows), predominantly on the left, consistent with retrobulbar optic neuritis (normal fundoscopy expected in 60%-90% of MOGAD/NMOSD cases). The radiological report confirmed chiasmal sparing (not pictured). This isolated retrobulbar pattern supports AQP4+ NMOSD over MOGAD, which characteristically features anterior perineural enhancement and optic disc edema. MRI: Magnetic resonance imaging; AQP4: Aquaporin-4; MOGAD: Myelin oligodendrocyte glycoprotein antibody disease; NMOSD: Neuromyelitis optica spectrum disorder

Brain MRI with and without contrast confirmed the extensive NMOSD distribution spanning the periventricular white matter through the area postrema to multilevel spinal cord LETM (C1-C6/T2-T6). Characteristic infratentorial T1/T2 lesions with normal DWI (Figure 1), dramatic short tau inversion recovery (STIR) resolution after treatment (Figure 2), the spinal H-sign amid lateral column involvement (Figure 3), and bilateral retrobulbar optic nerve enhancement despite normal fundoscopy (Figure 4) fulfilled the core diagnostic criteria while distinguishing NMOSD from MOGAD and MS mimics.

Re-interrogation revealed a seven-day persistent vomiting syndrome (APS) 1.5 months prior. Post-COVID-19 NMOSD was suspected. AQP4-IgG antibodies (cell-based assay (CBA)) were positive (1:10 dilution); antinuclear antibodies (ANAs) were positive at a titer of 1:80, anti-dsDNA antibodies were negative, and complement levels were normal. Anti-MOG antibodies were negative (evaluated via a commercial fixed CBA read by immunofluorescence; EU90-Euroimmun AG, Germany, as a live CBA was unavailable).

Methylprednisolone pulses (30 mg/kg/day for five days), followed by oral prednisolone (1 mg/kg/day for two months), improved gait completely but not vision. Five plasmapheresis exchanges achieved full neurological recovery. The patient was discharged after five weeks on azathioprine (2 mg/kg/day) and rituximab (700 mg IV, two doses every six months for two years). A control MRI at four months demonstrated complete lesion resolution (Figure 2C). At one year post-onset, the patient had received two doses of maintenance rituximab therapy and remained clinically relapse-free. A formal Expanded Disability Status Scale (EDSS) score and detailed long-term visual outcomes are not available.

## Discussion

NMOSD cases are a rare complication following SARS-CoV-2 infection, and this report contributes to the global knowledge of this pathology by detailing an atypical pediatric presentation. While the clinical course, radiological findings, and laboratory results of pediatric NMOSD generally mirror those of adults - typically featuring LETM - pediatric patients experience a lower frequency of optic neuritis, APS, and brainstem syndrome [1]. In the presented case, however, the extensive infratentorial involvement (LETM, APS, and brainstem syndrome) was a prominent feature, requiring careful differentiation from pediatric MS and MOGAD (Table 2) [5,9,16,17]. Accurate differentiation dictated the treatment strategy; while traditional MS therapies can exacerbate NMOSD, confirming AQP4+ NMOSD justified the initiation of lifelong immunosuppressive biologic therapy to prevent severe relapses.

**Table 2:** Differential diagnosis: pediatric MS, MOGAD, and NMOSD Comparison of key clinical, radiological, laboratory, and prognostic features. ADEM: Acute disseminated encephalomyelitis; BSIFE: Brainstem first episode; CBA: Cell-based assay; CIS: Clinically isolated syndrome; OCB: Oligoclonal bands; ON: Optic neuritis; VSS: Vertebral segments; MS: Multiple sclerosis; MOGAD: Myelin oligodendrocyte glycoprotein antibody disease; NMOSD: Neuromyelitis optica spectrum disorder; LETM: Longitudinally extensive transverse myelitis

Aspect	Pediatric MS	Pediatric MOGAD	Pediatric AQP4+ NMOSD
Incidence (per 100k/yr)	0.5-1 (adults 3-5)	0.3-0.4 (adults 3-4)	0.05-0.2 (adults 2)
Sex ratio (F:M)	2-3:1	1:1	1.5-3:1 (adults 9:1)
Other antibodies	ANA± (5-10%)	None	ANA+ 30-50%
Optic neuritis	Unilateral/short retrobulbar (~67%)	Bilateral/large anterior (25-50% retrobulbar)	Bilateral/longitudinal retrobulbar (~60-70%)
ON fundoscopy	Normal (2/3 cases)	Papilledema (50-75%); normal retrobulbar	Normal acute (60-70%)
Myelitis	Short peripheral (<2VSS)	Symmetric LETM (>3VSS)	Severe LETM (>3VSS), central grey
Area postrema	Rare (<5%)	Rare/None	20-40%
Brainstem syndrome	Common (20-30%), pontine	20-35%; multifocal, MCP/pons/medulla	12-57%; isolated + nausea/hiccups (56% BSIFE)
Brain MRI lesions	Periventricular, Dawson's, persistent	Fluffy/cortical/talamic, 60% resolution	Periventricular/postrema, 25% resolution
CSF OCB	70-90%	5-11%	20-40%
Rituximab response	Excellent (>90%)	Partial (50-70%)	Excellent (80-95%)
Acute treatment	Methylprednisolone ± IVIG/plasmapheresis	Methylprednisolone ± IVIG or plasmapheresis	Methylprednisolone + early plasmapheresis
Maintenance therapy	High-efficacy DMTs; relapse ↓>70%	AZA/mycophenolate/RTX; relapse ↓50-70%	RTX > AZA/mycophenolate
Recovery post-relapse	Good progressive	Good (monophasic 50%)	Poor
Disease course	Progressive, >100 lesions	Monophasic/good recovery	Relapsing, poor recovery

This case is highly atypical due to the patient's male sex (NMOSD F:M ratio is 1.5-9:1) and the specific presentation of retrobulbar optic neuritis (featuring mild MRI enhancement with a completely normal fundoscopy, a pattern seen in 60-70% of NMOSD cases). The AQP4-IgG-positive result is highly reliable, as it was processed using a CBA. While anti-MOG antibodies are statistically more frequent in pediatric demyelinating cohorts (11:1 compared with AQP4), the patient was confirmed to be MOG-negative via CBA. The clinical, radiological, laboratory, and therapeutic response data unequivocally support the isolated AQP4+ NMOSD diagnosis, aligning with the literature indicating that dual AQP4/MOG positivity is exceedingly rare (<0.1%). A monophasic course is unusual for NMOSD and is a hallmark of this report [3-5].

Considering the subgroup of NMOSD associated with SARS-CoV-2 infection, the review by Harel et al. [14] of 15 studies provides a valuable benchmark, although the presented case diverges in several demographic and clinical aspects. Harel et al.'s cohort demonstrated a strong female predominance (73%) and a mean age of 38 years (range, 7.5-71 years), with Latin American reports originating exclusively from Brazil; the current report, therefore, adds a rare pediatric male presentation from Colombia. In their review, the median interval between viral infection and NMOSD symptoms was 14 days, with 67% of the total cohort testing AQP4+ and 53% presenting with LETM. Focusing specifically on the four pediatric cases in that review (all girls aged 7.5 to 16 years, one with comorbid juvenile idiopathic arthritis), their SARS-CoV-2 serology and testing varied: two were asymptomatic (one PCR+ and one IgG+), one had typical COVID-19 symptoms (PCR+), and one lacked symptom data (IgG+/IgM+). The presented case directly mirrors the asymptomatic, isolated SARS-CoV-2 IgG+ serological profile seen in one of these girls. Regarding NMOSD serology, three of the four pediatric girls in Harel et al.'s review were AQP4-positive (aligning with the presented case), while the youngest (7.5 years old) was double-negative (AQP4-/MOG-). Notably, treatment responses in the literature diverged based on these serological profiles: the three AQP4+ girls improved with methylprednisolone alone, whereas the double-negative patient required triple therapy (methylprednisolone, plasmapheresis, and immunoglobulin). Strikingly, despite sharing the isolated AQP4+ serology of the steroid-responsive pediatric cases, the presented patient was highly resistant to initial high-dose steroids and required rapid escalation to plasmapheresis to achieve clinical improvement.

A review by Mirmosayyeb et al. [10] included a 15-year-old male who developed optic neuritis four weeks post-COVID-19 (PCR+). While demographically similar to the presented case, that patient was dual-positive for AQP4 and MOG antibodies, and diagnostic evaluation was limited solely to a brain MRI. Their patient responded well to methylprednisolone - a favorable steroid response and monophasic course typical of MOG positivity. This contrasts sharply with the isolated AQP4-positive disease documented here, which demonstrated severe steroid resistance. Furthermore, unlike MOG positivity, isolated AQP4+ NMOSD is notoriously relapsing (non-monophasic), necessitating the aggressive long-term biologic maintenance therapy utilized in this case.

Regarding vaccine-associated AQP4+ NMOSD, a systematic review by Harahsheh et al. [11] of 16 cases found that symptom onset occurred within a maximum of six weeks post-vaccination (mean of one week). Their cohort consisted entirely of adults (23-80 years) and predominantly women (69%), with 62% presenting with LETM and 20% featuring optic neuritis and/or APS. Furthermore, 31% had a history of immune-mediated conditions. While the extensive spinal cord and area postrema involvement in the presented case clinically mirrors these vaccine-triggered profiles (which share pathophysiological similarities with the non-vaccinated, post-infectious cases described by Harel et al. [14]), a vaccine etiology was confidently excluded here. The extensive two-year interval elapsed since this patient's COVID-19 vaccination far exceeds the established six-week window, ruling out causality.

A special consideration in this report is the patient's concurrent active dengue infection. Because dengue symptoms appeared only on the last day of the acute illness, the timeline indicates the virus did not act as the primary trigger for LETM-NMOSD. However, it is hypothesized that this concurrent viral infection contributed to the patient's inadequate steroid response. Previous literature describes NMOSD triggered by dengue in two adults and two adolescents, all of whom were AQP4+ and presented with optic neuritis and/or LETM [18]. In those pediatric reports, an 11-year-old improved with initial steroids, and a 17-year-old experienced a recurrence that successfully resolved with just a second course of methylprednisolone. Conversely, the concurrent dengue infection in the presented case was associated with severe steroid refractoriness.

Consequently, management aligned with standard escalation protocols [1,5]. Because the patient exhibited an inadequate response to high-dose IV methylprednisolone within the first five days, treatment was promptly escalated to plasmapheresis (five cycles), which successfully halted the inflammatory cascade. Preventive maintenance therapy was subsequently established using azathioprine and rituximab, resulting in complete clinical and radiological resolution at follow-up and underscoring the efficacy of this stepwise approach in severe pediatric NMOSD.

## Conclusions

This pediatric case of AQP4-IgG-positive NMOSD provides specific clinical lessons for managing severe demyelinating events temporally associated with COVID-19. First, it underscores that intractable vomiting must be recognized as APS, a critical sentinel presentation in atypical demographics such as pediatric males. Second, it highlights that a normal fundoscopy does not rule out optic neuritis; early MRI is essential to identify isolated retrobulbar enhancement and concurrent brainstem disease, aiding differentiation from MOGAD via anti-MOG testing. Finally, early CBA testing for AQP4-IgG proved crucial; given that AQP4-positive disease is associated with worse outcomes compared with MOGAD, aggressive therapeutic escalation (plasmapheresis and rituximab) in steroid-refractory cases was associated with neurological recovery.
